# From the Global Initiative for Asthma report and asthma guidelines to real-life asthma control: is there room for improvement?

**DOI:** 10.1186/s13052-022-01304-8

**Published:** 2022-07-05

**Authors:** Elio Novembre, Mattia Giovannini, Simona Barni, Francesca Mori

**Affiliations:** Meyer Children’s University Hospital, Viale Pieraccini 24, 50139 Florence, Italy

**Keywords:** Global Initiative for Asthma, Real-life, Improvement, Asthma, Pediatrics

## Abstract

Available guidelines for asthma management represent an important and suitable tool to make the entire medical process evidence-based, effective, and safe for patients. Their purpose is to help doctors and patients formulate the best decisions in regard to asthma management by choosing the most appropriate strategies in each specific clinical situation.

The Global Initiative for Asthma (GINA) document, together with other national and international recommendations, is one of the main documents used for asthma prevention and management in Italy, but several studies reported that these recommendations are often not applied in real-life clinical practice, which consequently results in inadequate asthma control.

In this context, a substantial simplification of the GINA document and asthma guidelines may represent a feasible strategy to be pursued to ameliorate the knowledge among GPs, primary care pediatricians, and specialists taking care of children and adults with asthma.

On the other hand, another critical factor that may explain unsatisfactory control of asthma is the limited importance that all recommendations place on asthma heterogeneity. In the era of personalized medicine and target therapies, phenotype-driven asthma management may become a desirable approach for optimizing the management of asthmatic patients. In addition, digital health strategies have been investigated in the literature to improve asthma monitoring and may represent a promising tool in the future from this point of view.

Relevant stakeholders should continue to investigate how to optimize real-life asthma control to propose novel solutions to translate into clinical practice.

To the Editor

In Europe, asthma affects about 30 million children and adults under 45 years of age, with a prevalence in Northern and Western countries. It ranges from less than 3% to more than 9% among adults aged 18–44. [1]. In Italy, asthma prevalence occurs at a rate of about 7% among the general population [2] and at rates of 9.5% and 10.4% among children and adolescents, respectively [3].

Available guidelines for asthma management represent an important and suitable tool to make the entire medical process evidence-based, effective, and safe for patients; their purpose is to help doctors and patients formulate the best decisions in regard to asthma management by choosing the most appropriate strategies in each specific clinical situation.

The Global Initiative for Asthma (GINA) was established through a 1993 collaboration between the World Health Organization and the National Heart, Lung, and Blood Institute (National Institutes of Health) to develop an asthma prevention and management strategy. This plan is updated yearly in a report based on the latest scientific evidence, and it is not defined as a guideline.

The GINA report describes asthma as a heterogeneous disease that is usually characterized by chronic airway inflammation. It is distinguished by a history of respiratory clinical manifestations such as wheezing, shortness of breath, chest tightness, and coughing that vary over time and in intensity, along with a variable expiratory airflow limitation.

The GINA document, together with other national and international recommendations, is one of the main documents used for asthma prevention and management in Italy, but several studies reported that these recommendations are often not applied in real-life clinical practice, which consequently results in inadequate asthma control [4].

Even in other developed European countries, a high percentage of patients (40–50%) have uncontrolled asthma; such patients use oral corticosteroids at an increased rate, and their condition can lead to emergency department visits and hospitalizations [5].

It was reported in Italian children and adolescents, that well-controlled asthma was achieved in 55% of patients, but 32.4% had partly controlled asthma, and 12.6% had uncontrolled asthma [6].

Moreover, in a large Italian study, 7264 children were diagnosed as asthmatic by 93 primary care pediatricians (72.9% with intermittent asthma, 25.4% with persistent asthma, and 1.7% with severe asthma) [7]. The asthma severity was measured according to the GINA document. Concerning intermittent asthma, 45% of participants used low-dose inhaled corticosteroid (ICS), 28% used bronchodilators on demand, 16% used anti-leukotriene drugs, and 11% referred the patient to the specialist.

In another recent study [8], 995 asthmatic patients with a mean age of 43.3 ± 17.7 years were enrolled by 107 Italian general practitioners (GPs) distributed throughout the country. Data on diagnosis, disease severity, prescribed drugs, and asthma control were collected through questionnaires filled out by GPs taking into consideration the GINA document. It was found that 39.7% of the patients with intermittent asthma were prescribed ICS plus long-acting beta-agonists independently from their signs and symptoms in the past year, thus risking potential overtreatment. In general, a low adherence (28.8%) of the GPs to the GINA report treatment recommendations was found.

All these recent data confirm previous reports that state that frequently, doctors do not correctly follow evidence-based practices [9] with no apparent improvement over time. Indeed, unsatisfactory physician adherence to asthma guidelines was reported in two surveys in 2010 and in 2019 [8, 10].

Moreover, recommendations are partially followed by specialists. Indeed, treatment intensity was not increased by pulmonologists in uncontrolled or partly controlled patients, and it was modified in only 37.2% of patients with a mean age of 46.9 ± 19.2 years [11].

Therefore, it appears that not only that the recommendations are not frequently followed by GPs, primary care pediatricians, and specialists, but inappropriate use of steroids was recently reported in Italy in both children [7] and adults [8], mainly in intermittent asthma patients.

Failure in asthma control can usually be considered the result of a complex interaction among different variables, e.g., the role of asthma guidelines diffusion and implementation, some disease-related factors (e.g., the presence of common comorbidities in asthma such as gastroesophageal reflux disease, sleep disturbances and obstructive sleep apnea, and rhinitis) or patient-related factors (e.g., adherence to treatment, alexithymia, and coping strategies).

In particular, the role of the GINA document and asthma guidelines diffusion and implementation must be underlined. Different factors may influence the process, some linked to the asthma guidelines themselves (e.g., complexity, degree of evidence, and transparency), and some to their implementation (e.g., communication strategies, educational techniques, and use of incentives). Moreover, socio-cultural context (e.g., standard of practice, social and clinical environments and habits) may represent a barrier that limits the achievement of the asthma guidelines’ goals and, therefore, the improvement of asthma control [12].

In this context, a substantial simplification of the GINA document and asthma guidelines may represent a feasible strategy to be pursued to ameliorate knowledge among GPs, primary care pediatricians, and specialists taking care of children and adults with asthma.

On the other hand, another critical factor that may explain unsatisfactory control of asthma is the limited importance that all recommendations place on asthma heterogeneity. In the era of personalized medicine and target therapies, phenotype-driven asthma management may become a desirable approach for optimizing the management of asthmatic patients [13, 14]. In addition, digital health strategies have been investigated in the literature to improve asthma monitoring and may represent a promising tool in the future from this point of view [15].

Relevant stakeholders should continue to investigate how to optimize real-life asthma control to propose novel solutions to translate into clinical practice.

## Data Availability

Not applicable.

## Abbreviations

GPs: General practitioners
GINA: Global Initiative for Asthma
ICS: Inhaled corticosteroid

## References

[1] Gibson GJ, Loddenkemper R, Lundbäck B, Sibille Y (2013). Respiratory health and disease in Europe: the new European Lung White Book. Eur Respir J.

[2] Maio S, Baldacci S, Carrozzi L, Pistelli F, Angino A, Simoni M (2016). Respiratory symptoms/diseases prevalence is still increasing: a 25-yr population study. Respir Med.

[3] Galassi C, De Sario M, Biggeri A, Bisanti L, Chellini E, Ciccone G (2006). Changes in Prevalence of Asthma and Allergies Among Children and Adolescents in Italy: 1994–2002. Pediatrics.

[4] de Marco R, Cazzoletti L, Cerveri I, Corsico A, Bugiani M, Accordini S (2005). Are the Asthma Guideline Goals Achieved in Daily Practice? A Population-Based Study on Treatment Adequacy and the Control of Asthma. Int Arch Allergy Immunol.

[5] Price D, Fletcher M, van der Molen T (2014). Asthma control and management in 8,000 European patients: the REcognise Asthma and LInk to Symptoms and Experience (REALISE) survey. NPJ Prim Care Respir Med.

[6] Licari A, Ciprandi G, Marseglia GL, Silvestri M, Tosca MA, Anastasio E (2020). Asthma in children and adolescents: The control’asma project. Acta Biomed.

[7] Tosca MA, Schiavetti I, Duse M, Marseglia GL, Ciprandi G, Licari A (2021). A Survey on the Management of Children with Asthma in Primary Care Setting in Italy. Pediatr Allergy Immunol Pulmonol.

[8] Baldacci S, Simoni M, Maio S, Angino A, Martini F, Sarno G (2019). Prescriptive adherence to GINA guidelines and asthma control: An Italian cross sectional study in general practice. Respir Med.

[9] Baiardini I, Braido F, Bonini M, Compalati E, Canonica GW (2009). Why do doctors and patients not follow guidelines?. Curr Opin Allergy Clin Immunol.

[10] Braido F, Baiardini I, Stagi E, Piroddi MG, Balestracci S, Canonica GW (2010). Unsatisfactory asthma control: Astonishing evidence from general practitioners and respiratory medicine specialists. J Investig Allergol Clin Immunol.

[11] Braido F, Baiardini I, Alleri P, Bacci E, Barbetta C, Bellocchia M (2017). Asthma management in a specialist setting: Results of an Italian Respiratory Society survey. Pulm Pharmacol Ther.

[12] Braido F (2013). Failure in Asthma Control: Reasons and Consequences. Scientifica (Cairo).

[13] Pérez de Llano L, Dacal Rivas D, Blanco Cid N, Martin Robles I (2021). Phenotype-Guided Asthma Therapy: An Alternative Approach to Guidelines. J Asthma Allergy.

[14] Giovannini M, Mori F, Barni S, de Martino M, Novembre E (2019). Omalizumab and mepolizumab in the landscape of biological therapy for severe asthma in children: how to choose?. Ital J Pediatr.

[15] Mosnaim G, Safioti G, Brown R, Depietro M, Szefler SJ, Lang DM (2021). Digital Health Technology in Asthma: A Comprehensive Scoping Review. J Allergy Clin Immunol Pract.

